# Developing an efficient DNA barcoding system to differentiate between *Lilium* species

**DOI:** 10.1186/s12870-021-03229-6

**Published:** 2021-10-13

**Authors:** Yixin Liu, Mingfang Zhang, Xuqing Chen, Xi Chen, Yue Hu, Junlian Gao, Wenqiang Pan, Yin Xin, Jian Wu, Yunpeng Du, Xiuhai Zhang

**Affiliations:** 1 Beijing Academy of Agriculture and Forestry Sciences, Beijing, 100097 China; 2 Beijing Key Laboratory of Development and Quality Control of Ornamental Crops, Department of Ornamental Horticulture and Landscape Architecture, China Agricultural University, Beijing, 100193 China; 3 School of Landscape Architecture, Beijing Forestry University, Beijing, 100083 China

**Keywords:** *Lilium*, cpDNA hypervariable region, DNA barcoding, Species identification, Phylogenesis

## Abstract

**Background:**

*Lilium* is an important ornamental bulb, possesses medicinal properties, and is also edible. Species within the *Lilium* genus share very similar morphology and macroscopic characteristics, thus they cannot be easily and clearly distinguished from one another. To date, no efficient species-specific markers have been developed for classifying wild lily species, which poses an issue with further characterizing its medicinal properties.

**Results:**

To develop a simple and reliable identification system for *Lilium*, 45 representative species from 6 sections were used to develop a DNA barcoding system, which was based on DNA sequence polymorphisms. In this study, we assessed five commonly used DNA barcode candidates (*ITS*, *rbcL*, *ycf1b*, *matK* and *psbA-trnH*) and five novel barcode candidates obtained from highly variable chloroplast genomic regions (*trnL-trnF*, *trnS-trnG*, *trnF-ndhJ*, *trnP-psaJ-rpI33* and *psbB-psbH*). We showed that a set of three novel DNA barcodes (*ITS + trnP-psaJ-rpI33 + psbB-psbH*) could be efficiently used as a genetic marker to distinguish between lily species, as assessed by methods including DNAsp, BI and ML tree, and Pair Wise Group (PWG).

**Conclusions:**

A rapid and reliable DNA barcoding method was developed for all 45 wild *Lilium* species by using *ITS*, *trnP-psaJ-rpI33*, and *psbB-psbH* as DNA barcoding markers. The method can be used in the classification of wild *Lilium* species, especially endangered species, and also provides an effective method for selective lily breeding.

**Supplementary Information:**

The online version contains supplementary material available at 10.1186/s12870-021-03229-6.

## Background

*Lilium* is a genus of flowering plants which includes 110-115 species of lily. Lilies are endemic to the cold and temperate regions of the northern hemisphere [1–4]. Lilies have originated from the Himalayas and the southwest of China, where there are currently about 51 species/varieties present [5, 6]. Based on their morphological classification, these species are divided into the following 8 sections: Section *Martagon*, Sect. *Pseudolirium*, Sect. *Liriotypus*, Sect. *Archelirion*, Sect. *Sinomartagon*, Sect. *Leucolirion*, Sect. *Lophophorum*, and Sect. *Lilium—Nomocharis* [7]*.* Today, more than 10,000 cultivars have been registered in the Royal Horticulture Society, showcasing great value around the world [8]. Lily cultivars are also classified into 8 groups (Oriental hybrid, LA-hybrid, OT-hybrid, Asiatic hybrid, LO-hybrid, Longiflorum, and Aurelian & Trumpet), which are cultivated to create ornamental value [2, 4]. *Lilium* species are not only used as ornamental plants but also to produce food and medicine, especially in Asia. The traditional edible lilies mainly include *L. davidii* var. *willmottiae*, *L. brownii*, *L. lancifolium*, *L. longiflorum* [9]. Moreover, *L. lancifolium*, *L. pumilum* and *L. brownii* are used as a component of traditional Chinese medicine for lung ailments [10, 11]. Due to their ornamental, edible, and medicinal properties, *Lilium* species have great commercial value [9].

However, it is difficult to clearly identify *Lilium* species within the same category solely based on their highly similar morphological characteristics [12–14]. Additionally, as a result of overharvesting and reduction in their natural habitat, the abundance of wild lily species has dramatically decreased [9, 15, 16]. This has led to several species being listed as key protected wild plants, including *L. fargesii*, *L. amoenum*, *L. henrici*, *L. paradoxum*, *L. taliense*, and *L. wardii* [17–20]. Hence, the ability to correctly identify endangered lily species using genetics may serve a role in effectively protecting these species. Furthermore, the high species diversity found within the same ecosystem further muddles our process to correctly identify lily species based on traditional morphology-based taxonomy, resulting in ambiguous phylogenetic classification. DNA marker technology, such as the use of DNA barcoding in classification systems, has significantly enhanced the identification, protection, and sustainable use of plant resources. However, no high-resolution markers for *Lilium* have been developed yet.

DNA barcoding is a system that involves the sequencing of short DNA fragments for fast and accurate species identification, especially suitable for highly homologous species [21, 22]. DNA barcoding is a widely used method for distinguishing macroscopically similar species, detecting the spatial distribution of plant roots, and studying invasive plant species [22]. Moreover, the genetic sequences used in DNA barcoding can be conserved enough across species to facilitate the design of universal sequencing primers. DNA barcoding is also beneficial to the fields of conservation and evolutionary ecology [23], where this method is utilized to evaluate the genetic diversity of endangered species needed for their protection and population restoration [24]. Universal barcodes, such as *rbcL*, *matK*, and *psbA-trnH*, use sequences from chloroplast genes and have been previously identified in different species [25–30]. However, apart from universal barcodes, we also require the development of species-specific DNA barcodes [31–33].

With the rapid development of next-generation sequencing, obtaining chloroplast-specific genomic sequences has become much easier and can be used in extending gene-based phylogenetics to phylogenomics [34, 35]. Differences in chloroplast (cp) genomic sequences between plant species can be comprehensively applied in the phylogenetic classification of individual plants [36]. While the cp genome contains highly conserved regions, the highly variable genomic regions could be used to identify DNA barcoding candidates which would resolve the phylogenetic relationship between species.

Recently, molecular phylogenetic studies in *Lilium* were carried out using molecular markers like RAPD (Random Amplified Polymorphic DNA) and ISSR (Inter-simple Sequence Repeat), which have low rates of PCR amplification and low sequencing resolution [39, 40]. In ideal conditions, a barcode should be variable enough to resolve closely related species and short enough for easy experimental manipulation with low cost. Therefore, in this study, we investigated the effectiveness of using DNA barcoding to distinguish between 45 representative *Lilium* interspecies and developed an efficient DNA barcoding system by using a combination of genetic markers (*ITS*, *trnP-psaJ-rpI33*, and *psbB-psbH*).

## Results

### Hypervariable region assessment within the chloroplast genome in *Lilium spp.*

To identify potential DNA barcodes, we screened previously published genomic data from 16 *Lilium* chloroplast (cp) genomes for hypervariable regions [19]. By using multiple sequence alignment, we identified the following hypervariable regions: *trnS-trnG, trnE-trnT-psbD, trnF-ndhJ, psbE-petL, trnP-psaJ-rpl33, psbB-psbH, petD-rpoA, ndhF-rpl32-trnL, ycf1a, and ycf1b* [19]*.* A total of 521 nucleotide polymorphisms were identified in these 10 regions, which may be used as genetic markers for phylogenetic construction and species identification in *Lilium*. In order to evaluate the nucleotide polymorphism information (Pi), the stepwise genetic nucleotide diversity (π; Nei 1987) was estimated within the 10 regions. The π, representing the existence of different nucleotide bases between species, ranged from 0.01364 to 0.01833 within the 16 species. This data indicates the presence of mutations in the hypervariable regions of the relatively stable chloroplast genome, which could be used to develop candidate DNA barcodes [19].

We then successfully amplified five of the ten hypervariable regions by PCR. To develop high-resolution DNA barcoding, we added another five canonical plant barcoding markers (Table 2). Among the candidate barcodes, *psbA-trnH* was the shortest in length (400 bp) and *psbB-psbH* was the longest (1124 bp), with an average size of 727 bp. Ten DNA barcodes were then used to amplify sequences from 45 species used, plus an additional three outgroups (*Cardiocrinum giganteum*, *Nomocharis pardanthina*, and *Fritillaia karelinii*). This resulted in the amplification of 472 different sequences. The sequencing success rate of *trnF-ndhJ* (93.7%) and *ITS* (91.6%) were relatively low, while the rest of the DNA barcodes had 100% sequencing success rates (Table 4).

## Barcoding assessment using chloroplast and nuclear markers in *Lilium spp.*

To assess the efficiency of the DNA barcodes identified, we evaluated eight different barcoding indices to assess the potential for each candidate sequence to be useful in lily DNA barcoding. The total number of mutations (Eta), haplotype diversity (Hd), π, G + C ratio, information site, and average nucleotide difference (k), were analyzed for all 472 amplified DNA sequences (Table 3). The Hd index was highest for the following genomic sequences: *ITS* (0.995), *trnF-ndhJ* (0.965), *ycf1b* (0.952), *matK* (0.94), and *psbB-psbH* (0.926). This data shows that these genomic regions would have the highest allelic difference in randomly sampled individuals and could thus serve as a useful parameter in distinguishing individual species. We then selected those five regions for further analysis to determine their potential as DNA barcodes. The genetic nucleotide diversity (π) was high, ranging from 0.03035 to 0.30907. The total number of mutations (Eta) of the five candidate barcodes was also higher compared to other sequences (244 to 372, except for *trnF-ndhJ* with an Eta of 49). Notably, the lowest overall scores were mainly given by the analysis of the conserved DNA barcodes. For example, *psbA-trnH* had a Hd score of 0.792. Taken together, all the above analyses show that we identified five possible barcoding regions: *trnP-psaJ-rpI33*, *psbb-psbh*, *ycf1b*, *ITS,* and *matK*.

**Fig. 1:**
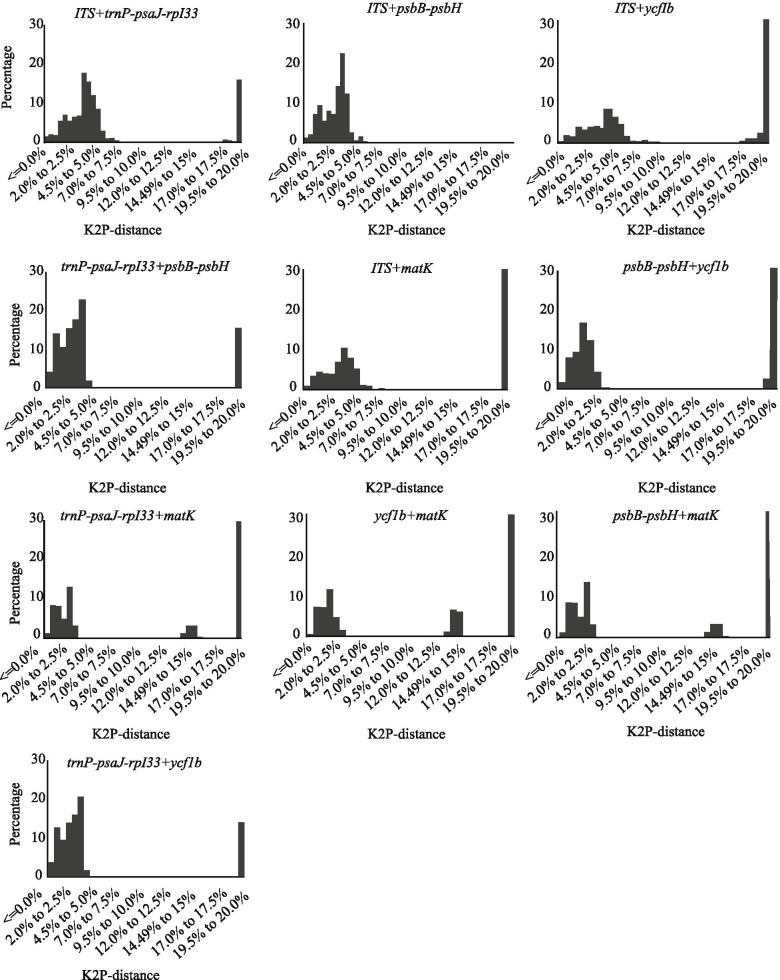
Barcoding gap assessment for two candidate barcodes combinations. x-axes relate to K2P distances and y-axes represent the percentage of occurrences

**Fig. 2:**
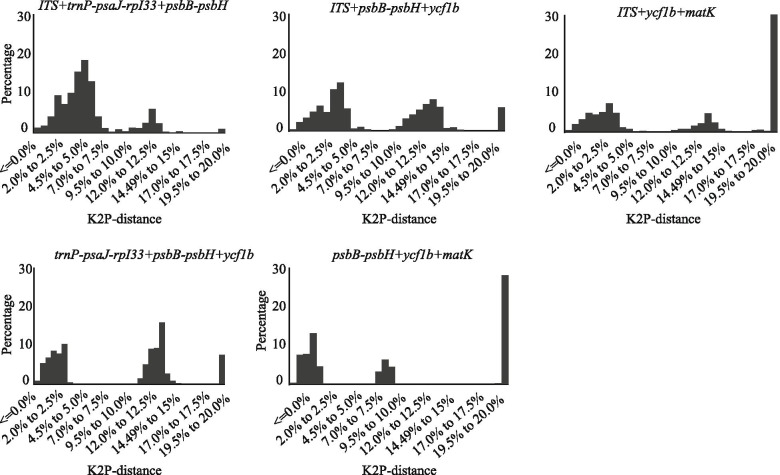
Barcoding gap assessment for three candidate barcodes combinations. x-axes relate to K2P distances and y-axes represents the percentage of occurrences

**Fig. 3:**
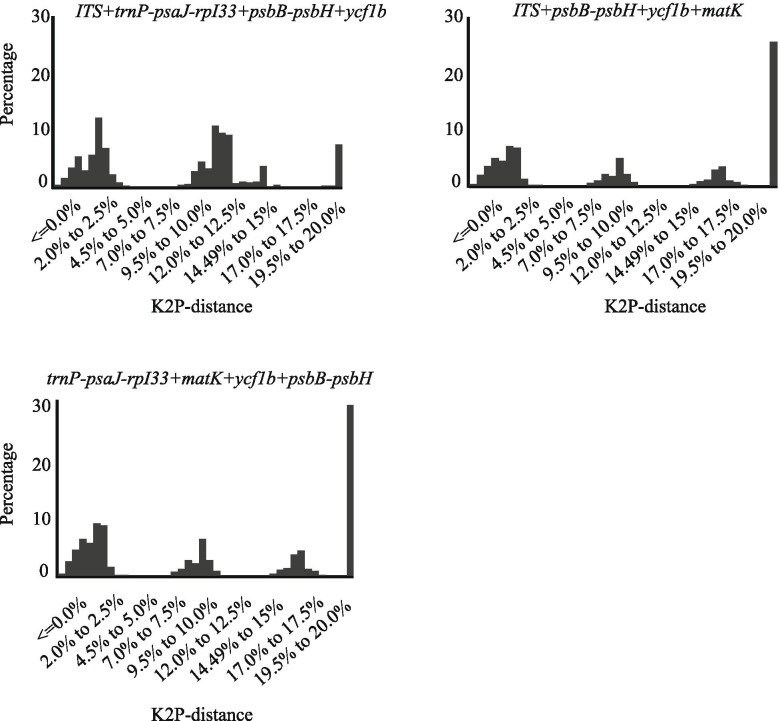
Barcoding gap assessment for four candidate barcodes combinations. x-axes relate to K2P distances and y-axes represents the percentage of occurrences

**Fig. 4:**
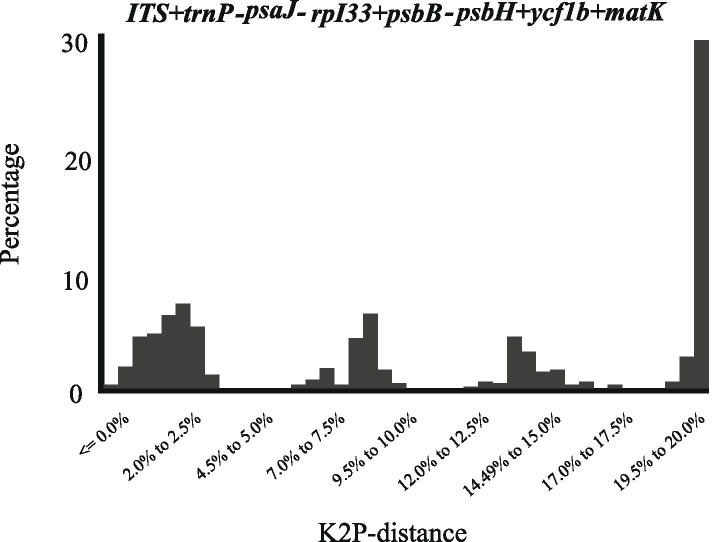
Barcoding gap assessment for five candidate barcodes combinations. x-axes relate to K2P distances and y-axes represents the percentage of occurrences

To estimate the genetic diversity between interspecies in *Lilium*, these five candidate barcodes were chosen for combinatorial barcode analysis by Kimura-2-Parameter (K2P). Overall, 19 combinations of candidate barcodes were obtained, and the results showed that 1) there were no barcode gaps when combining two barcodes (Fig. 1); 2) the highest variation in interspecific divergence resulted from the combination of three barcodes (*ITS* + *trnP-psaJ-rpI33* + *psbB–psbHI*) (Fig. 2); and 3) the interspecific differentiation was relatively low, and the diversity could not be clearly observed when combining four or five barcodes (Figs. 3 and 4). These data demonstrated that a combination of three candidate barcoding sequences could be the most efficient in distinguishing between lily species**.**

### *Lilium spp.* identification using DNA barcoding

To evaluate the barcoding gap in comparing with the distribution of the pair-wise interspecific distance for single/ combined barcode(s), we analyzed DNA barcoding sequences using Taxon DNA. The ‘Best match’ analysis was then performed to determine the closest barcode match for given sequences, regardless of the sequence similarity in the barcoding sequence. This meant that every queried sequence would be assigned the best matching barcode. When comparing the results of the ‘Best match’ analysis and ‘Best close match’ analysis, the former always presented higher or equal individual identification rates (Table 4). The barcode index of combined candidates was generally higher than that of a single candidate barcode. This analysis demonstrates that the barcoding combination of *ITS + trnP-psaJ-rpI33 + psbB-psbH* has the highest potential success rate (12.33%) in correctly identifying the lily species, followed by*ITS+trnP-psaJ-rpI33*(10.41%).

## Tree-based analysis with chloroplast and nuclear DNA barcoding in *Lilium spp.*

**Fig. 5:**
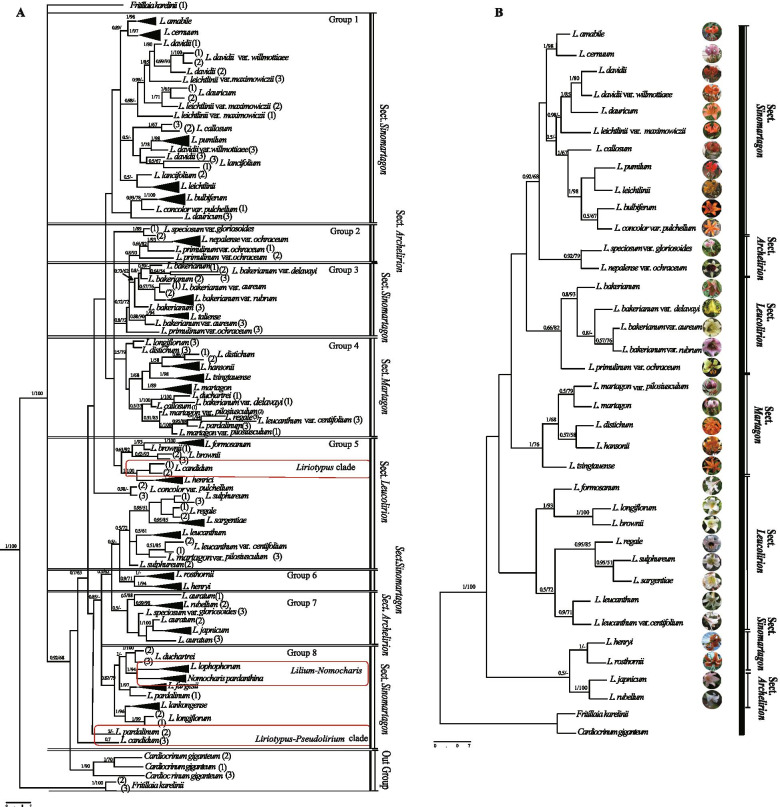
Phylogenetic tree interspecies verification. The maximum likelihood (**A**) and Bayes (**B**) phylogeny diagrams constructed from candidate barcodes verify the taxonomic relationship between *Lilium*. The number above the node is the support value, the right side is the ML guide value, and the left side is the Bayesian posterior probability (PP) value

To validate the resolution for using *ITS+trnP-psaJ-rpI33+psbB-psbH* as DNA barcoding to identify *Lilium* species, we verified that the candidate barcodes can clearly distinguish between species by constructing a phylogenetic tree. We used 45 representative species with three biological replicates each, by using maximum likelihood (ML) and Bayesian (BI) Phylogenetic tree. The results from both the ML and BI tree analyses divided these species into four sections and eight groups (Fig. 5). The four sections were Sect. *Sinomartagon*, Sect. *Archelirion*, Sect. *Martagon*, and Sect. *Leucolirion*. Within the Sect. *Sinomartagon*, the resulting identity was 96%, so this category was further divided into four groups: Group 1, Group 3, Group 6, and Group 8. A similar analysis was carried out within the Sect. *Archelirion*, where the species were divided into two groups, Group 2 and Group 7. Sect. *Leucolirion* only had one group (Group 5) and Sect. *Archelirion* mainly belonged to Group 4. The discrimination rate in Group 8 was 86%. Additionally, *Nomocharis pardanthina* and *L. lophophorum* in Group 8 clustered together in the same branch with an approval rating of [BI=1, ML=94], indicating that *N. pardanthina* was genetically closer to *Lilium*. Overall, the data validated the use of the newly identified DNA barcodes in accurately distinguishing species of *Lilium*.

## Discussion

*Lilium* is one of the most valuable ornamental plant genera worldwide, with 100-115 species and over 10,000 cultivars. However, until now, an efficient method for distinguishing *Lilium spp*. had yet to be established. Here, we developed and validated a set of high-resolution DNA barcodes for use as a tool in distinguishing 45 species representing six sections.

## Discrimination comparison among different analysis methods

Previously, several methods were generally used for analyzing interspecies discrimination with DNA barcoding, including phylogenetic trees (NJ, BI, MP, ML and et al.), distance-based (PWG, P-distance, K2P distance, and et al.), and character-based methods (BLOG, DNABAR, BRONX, and et al.) [41, 42]. However, there was no standard method in place for species identification [43, 44]. In this study, we used various methods to develop DNA barcoding specifically for species within the *Lilium* genus.

First, we used Taxon DNA analysis (simple pairwise matching for DNA barcoding) to evaluate the *Lilium*. The index from ‘Best match’ in Taxon DNA analysis was lower than those from the tree-based analysis. Thus, tree-based analysis was more suitable in determining DNA barcoding sequences for identifying different lily species. In Taxon DNA analysis, small barcoding gaps, high similarity in interspecies, unclear origin and obscure evolution contributed to low discrimination [19]. Next, we verified the candidate DNA barcodes by using BI and ML methods to construct the phylogenetic tree. To find a more accurate model, ML was repeated one thousand times and BI tree was repeated one billion times, which generated a more definite analysis than an NJ tree [45]. Then, we performed phylogenetic tree analysis to evaluate the identified candidate DNA barcodes in *Lilium* and revealed that Sect. *Sinomartagon* and Sect. *Archelirion* were more differentiated than the other sections. In the Group 8, *N. aperta* was close to *N. pardanthina* [46] but nested within *Lilium*, which was clustered in the same branch as the *L. duchartrei* of Sect. *Sinomartagon* clustered. Overall, the genetic analysis of DNA barcodes gave similar classification results compared to morphological classification, which demonstrates the reliability of the recommended barcodes. However, using DNA barcodes to identify between species is a superior classification method to using morphological characteristics. Using DNA barcoding for successful identification at the subgenus or node level could also be considered if individuals form a monophyletic clade [19]. Additionally, the appropriate phylogenetic methodology could provide a reliable reference for the study of the origin, phylogenetic, and differentiation ages, and help solve the phylogenetic relationship and classification complications in *Lilium.*

## The Evaluation of *Lilium* DNA Barcode

In this study, hypervariable regions of *Lilium* chloroplast genome and conserved plant fragments were selected as candidate DNA barcodes. An ideal DNA barcode requires clear species discrimination and high-quality primer pairs [47, 48]. According to the criteria above, we used eight indices (Table 3) to evaluate the candidate barcode index and five of those fifteen barcodes showed the highest indices quality and bidirectional sequences. The five identified candidate DNA barcodes were *matK*, *trnP-psaJ-rpI33, psbB-psbH, ycf1b*, and *ITS*. *ITS* is the only DNA sequence that belongs to a conserved ribosomal DNA genetic region, while the others belong to the hypervariable regions of cp genome DNA in the plant. This indicated that the mutation information in the hypervariable regions of the relatively stable chloroplast genome was suitable for developing candidate DNA barcodes for *Lilium spp.* identification.

As previous studies have reported, due to its high level of sequencing success rate (91.6%), the haplotype diversity (0.995), and the number of haplotypes (40), *ITS* provided the highest species resolution. *ITS* also has the highest species resolution as measured by “Best match (9.09%)”. Therefore, *ITS* is regarded as a suitable candidate for plant DNA barcoding and has been widely used in community phylogeny and biodiversity surveys [49]. We obtained similar results in *Lilium,* where *ITS* barcoding indices were significantly higher than those of other candidate DNA barcodes (Table 3, Table 4). To our excitement, we found that *trnP-psaJ-rpI33* and *psbB-psbH* yielded the most promising results as DNA barcodes since this combination had a sequencing success rate of 100% (Table 3). Furthermore, several reports have been previously published regarding the use of *matK* in DNA barcoding*,* which belongs to the conserved cp genetic regions [21, 50], and has thus led some researchers to have reservations about the use of this locus. In the current study, the *matK* locus was a relatively high information locus (192) and the success rate of sequencing using this locus was 100%, These data made *matK* as one of the candidate DNA barcodes in *Lilium*. We also noticed that the *matK* sequence was easy to amplify but it often performed poorly in complex evolving groups [51], which was consistent with previous reports. Overall, *ITS*, *trnP-psaJ-rpI33*, and *psbB-psbH* were the best single-barcode candidates for *Lilium identification*.

The use of a combination of barcodes can often improve the ability to identify species [52]. Of the 19 barcode combinations tested, *ITS + trnP-psaJ-rpI33 + psbB-psbH* performed best in the ‘Best match (12.33%)’, ‘Best close match (12.33%)’ and ‘all species barcodes (8.08%)’. The results of the combination of the two barcodes showed (Fig. 1) that combinations containing *ITS* significantly improved the resolution of species identification. *ITS + trnP-psaJ-rpI33* (10.41%) and *ITS + psbB-psbH* (8.33%) had higher discriminatory power than *psbB-psbH + matK* (6.25%) and *ycf1b + matK* (4.16%). In the analysis of three-barcode combinations (Fig. 2), we found that *ITS+psbB-psbH+ycf1b* (8.33%) has the same "Best Match” index as *ITS+psbB-psbH* (8.33%). Surprisingly, we were not able to increase the resolution success by combining four DNA barcodes. This may be related to the complexity of *Lilium* genome and the low resolution in distinguishing between sequences found within the conserved fragments. Considering discriminatory power, cost-efficiency and effort, the three-marker combination ‘*ITS+trnP-psaJ-rpI33+psbB-psbH*’ showed the best species identification among all the compared marker combinations, suggesting that it may be the best choice for barcoding in *Lilium*. Although various barcodes have been widely used in different plants before, the species classification within a specific genus is affected by many factors, which often results in the uncertainty of a single genetic site. Therefore, in order to develop an appropriate plant DNA barcoding, selected markers should not emphasize universality in all plant species but should be more specific for a certain taxon.

## Conclusions

In summary, lily is a highly valuable ornamental and medicinal plant. In this study, by constructing the phylogenetic tree, we identified that a combination of three DNA barcodes was the most effective method for differentiating lily species. The DNA barcodes were obtained from hypervariable as well as conserved regions within the chloroplast genome of 45 *Lilium* species. The development of DNA barcodes will provide an effective tool for the conservation of wild *Lilium* resources, the identification of edible and medicinal resources, and the development of new germplasms.

## Methods

### Plant materials

All plant materials are original from the National Lily Germplasm Bank at Beijing Academy of Agricultural and Forestry Sciences. To capture high-resolution genetic diversity, samples were collected in largescale and extensive distribution. Our endemic wild *Lilium* belonging to 34 species and 11 varieties (45 germplasm resources) of 6 sections were selected. The 6 sections include Sect. *Martagon*, Sect. *Archelirion*, Sect. *Sinomartagon*, Sect. *Leucolirion*, Sect. *Pseudolirium*, and Sect. *Liriotypus*. The section and quantity of each sample were listed in Table 1. Fresh leaves were sampled and stored at -80°C until DNA extraction. Total genomic DNA was extracted using a plant genome extraction kit (Tiangen, Beijing, China). Three samples of *Cardiocrinum giganteum*, *Nomocharis pardanthina*, and *Fritillaia karelinii* were used as outgroups.

**Table 1:** Information about tested *Lilium* species in this study

Section (Comber)	No. of tested species	Species
Sect. *Martagon*	6	*L. martagon , L. distichum, L. tsingtauense, L. hansonii, L. martagon* var*. pilosiusculum, L. henrici*
Sect. *Archelirion*	5	*L. rubellum, L. speciosum* var. *gloriosoides, L. auratum, L. japnicum, L. nepalense* var*.ochraceum*
Sect. *Sinomartagon*	20	*L. amabile, L. davidii , L. cernuum, L. leichtlinii* var. *maximowiczii, L. leichtlinii, L. dauricum, L. davidii* var.*willmottiaee, L. duchartrei, L. taliense, L. lankongense, L. callosum, L .fargesii, L.lophophorum, L. primulinum* var*.ochraceum, L. bulbiferum, L. pumilum, L. concolor* var. *pulchellum, L. lancifolium, L. henryi, L. rosthornii*
Sect*. Leucolirion*	12	*L. sulphureum, L. regale, L. brownii, L. leucanthum, L. leucanthum* var. *centifolium, L. formosanum, L. sargentiae, L. longiflorum*, *L. bakerianum* var*.aureum, L. bakerianum* var*. delavayi, L.bakerianum* var.*rubrum, L. bakerianum*
Sect. *Pseudolirium*	1	*L. pardalinum*
Sect*. Liriotypus*	1	*L. candidum*

**Table 2:** Screening results of each barcode primer

Gene Name	Length (bp)	Primer Name	Forward primer	Reverse primer
*trnF-ndhJ*	855bp	LHV3	TGGATATAGACCTCCATTTTTGAG	GATAATGACACGACTCCAGAA
*trnS-trnG*	665bp	S1 /LHV1	CTCTCCCAACTCAAAATTG	CAGAATTATGAAAATTATAGCGT
*trnP-psaJ-rpI33*	633bp	S5/LHV5	ATCCTTGTCTTGTTTTCCAC	TTCTAACTMTCAATTATTCCTA
*psbB-psbH*	1124bp	LHV6	GGGTTGGTTCACTTTTGGGC	TCCACGGTCGAACTACCAGA
*ycf1b*	750bp	LHV10	ACCACCCGTTTGGCTTTTCT	CCATGCCCATTTCCGGTTTG
*matK*	800bp	matK	CGATCTATTCATTCAATATTTC	TCTAGCACACGAAAGTCGAAGT
*rbcL*	600bp	rbcL	ATGTCACCACAAACAGAGAC	TCACATGTACCCGCAGTAGC
*psbA-trnH*	400bp	psbA-trnH	ACTGCCTTGATCCACTTGGC	CGAAGCTCCATCTACAAATGG
*trnL-trnF*	700bp	trnL-trnF	CGAAATCGGTAGACGCTACG	ATTTGAACTGGTGACACGAG
*ITS*	750bp	ITS	GGAAGKARAAGTCGTAACAAGG	RGTTTCTTTTCCTCCGCTTA

**Table 3:** Analysis of different barcoding indices of *Lilium*

	Hd	H	Pi	k	Eta	G+C Ratio (%)	Information site	success rate of sequences
*matK*	0.94	25	0.2556	96.362	372	30.1-31.3	192	100%
*psbA-trnH*	0.792	14	0.00250	4.100	22	29.2-32.5	70	100%
*trnL-trnF*	0.914	24	0.00883	6.137	52	31.9-33.0	14	100%
*rbcL*	0.814	16	0.00376	2.273	22	43.8-44.1	10	100%
*trnS-trnG*	0.879	20	0.02245	8.036	57	20.7-22.4	20	97.9%
*trnF-ndhJ*	0.965	23	0.01652	8.295	49	27.6-29.1	27	93.7%
*trnP-psaJ-rpI33*	0.880	19	0.10146	34.902	229	28.0-30.6	195	100%
*psbB-psbH*	0.926	21	0.03035	13.961	268	34.8-36.5	107	100%
*ycf1b*	0.952	26	0.30907	80.975	270	27.6-32.0	162	100%
*ITS*	0.995	40	0.05201	31.776	244	58.3-64.0	105	91.6%

Note: haplotype diversity (Hd), number of haplotypes (H), nucleotide diversity (Pi), average number of nucleotide differences (k), total number of mutations (Eta).

**Table 4:** Recognition success index based on the program TaxonDNA

Region	Best match			Best Close match			All species barcodes		
	Correct(%)	Ambiguous(%)	Incorrect(%)	Correct(%)	Ambiguous(%)	Incorrect(%)	Correct(%)	Ambiguous(%)	Incorrect(%)
*ITS+trnP-psaJ-rpI33+psbB-psbH*	12.33	12.5	79.16	12.33	10.41	79.16	8.08	95.83	0
*ITS+trnP-psaJ-rpI33*	10.41	10.41	79.16	10.41	10.41	75	4.16	89.58	2.08
*ITS*	9.09	40.9	50	9.09	31.81	34.08	0	75	0
*ITS+psbB-psbH*	8.33	37.5	54.16	8.33	37.5	52.08	2.08	93.75	2.08
*trnP-psaJ-rpI33+matK*	8.33	27.08	64.58	8.33	27.08	60.41	0	95.83	0
*ITS+psbB-psbH+ycf1b*	8.33	8.33	83.33	8.33	6.25	83.33	4.16	93.75	0
*ITS+trnP-psaJ-rpI34+psbB-psbH+ycf1b*	8.33	2.08	89.58	8.33	0	89.58	4.16	91.66	2.08
*trnP-psaJ-rpI33+psbB-psbH+ycf1b+matK*	8.33	2.08	89.58	8.33	0	89.58	4.16	91.66	2.08
*ITS+ycf1b*	8.16	28.57	63.26	8.16	26.52	59.18	8.16	85.71	0
*ITS+matK*	6.25	22.91	70.83	6.25	20.83	70.83	0	97.91	0
*ycf1b*	6.25	66.66	27.08	6.25	64.58	27.08	6.25	79.16	12.5
*matK*	6.25	50	43.75	6.25	47.91	43.75	0	97.91	0
*trnP-psaJ-rpI33+ycf1b*	6.25	45.83	47.91	6.25	43.75	47.91	6.25	87.5	4.16
*psbB-psbH+ycf1b*	6.25	45.83	47.91	6.25	43.75	47.91	2.08	93.75	2.08
*psbB-psbH+matK*	6.25	37.5	56.25	6.25	37.5	52.08	0	95.83	0
*ITS+psbB-psbH+ycf1b+matK*	6.25	6.25	87.5	6.25	6.25	81.25	0	93.75	0
*ITS+ycf1b+matK*	6.12	10.2	83.67	6.12	10.2	79.59	0	95.91	0
*ITS+trnP-psaJ-rpI34+psbB-psbH+ycf1b+matK*	6.12	4.08	89.79	6.12	4.08	87.75	0	95.91	2.04
*ycf1b+matK*	4.16	35.41	60.41	4.16	35.41	54.16	0	91.66	3.08
*trnP-psaJ-rpI33*	4.16	56.25	39.58	4.16	56.25	39.58	8.33	83.33	8.33
*trnP-psaJ-rpI33+psbB-psbH*	4.16	58.33	37.5	4.16	56.25	37.5	2.08	93.75	2.08
*psbB-psbH+ycf1b+matK*	4.16	18.75	77.08	4.16	18.75	70.83	0	91.66	2.08
*trnP-psaJ-rpI33+psbB-psbH+ycf1b*	0	39.58	60.41	0	37.5	60.41	0	95.83	2.08
*psbB-psbH*	0	81.25	18.75	0	81.25	16.66	0	95.83	2.08

### Primer design and PCR amplification

The software Geneious.10 was used to design 22 pairs of primers (attached file 1: Table S1) [54]. Tissue samples were taken and the products were amplified by Sanger sequencing. The primer fragments with low polymorphism sites and failed sequencing were also removed to determine whether the primer was suitable for *Lilium*. PCR was performed in 25 μL volume consisting of 10 ng of genomic DNA, 0.2 μL of LA Taq (Takara), 2.0 μL of dNTP, 2.5 μL of 10×buffer, 1 μL of upstream and downstream primers (10 μmol/L stock) and ddH_2_O supplemented to 25 μL. Amplification was performed in Veriti Thermal Cycler (Applied Biosystems) with the following program: 3 minutes at 95°C for initial denaturation; denaturation 95°C, 30 seconds; 35 cycles consisting, annealing temperature 50°C - 56°C, 45 seconds, extension temperature 72°C, 1 minute, final 7-minute extension at 72°C, 4°C low temperature save. At least 10 replications of each species were sequenced in both sense and antisense directions. PCR products were separated by electrophoresis on 1.2 % Agarose Gel and visualized with GelRed stain (Biotium, USA).

### Sequence alignment and data analysis

To modify heterozygous loci including W, R, Y, S, K, and M, the sequencing results which referred to the Chromas sequence peak map were performed by BioEdit v.7 [55]. Proofreading sequences were sequenced using Geneious’s MAFFT v7 (10.1093/nar/gkf436) [56] to quantify sequence length and base composition. Aligned sequences were counted using MEGA v 6.0 for the sequence of variant information [57] The PHASE operation was performed with DNA SP v5. 1[58] (run-length = 10 000 iterations, burn-in = 100, thinning interval = 10) to calculate the total number of nuclear mutations (Eta), haplotype diversity (Hd), nucleotide diversity information (Pi), average nucleotide difference (k), and the number of haplotypes (H). Pi was the detection of the most essential genetic differences between different individuals, and it represented the existence of different bases between species (sliding window=800 bp, step=200 bp). H refers to the number of different haplotypes contained in all the sequences under study. Hd refers to the frequency of randomly selecting two different haplotypes from a sample. The population with high Hd indicated rich genetic resources. Eta was the site at which a mutation occurs on a base of a sequence. K was the total nucleotide variation /the number of individual samples. Based on similarity obtained from Taxon DNA software, the individual-level discrimination rates of all possible single and combination markers were tested under the K2P-corrected distance model [59]. Taxon DNA with ‘pairwise summary function’ was used to estimate the barcoding gap. To precisely analyze *Lilium* species, each barcode candidate was measured for appropriate identification proportion by ‘best match’, ‘best close match’, and ‘all species barcode’ functions of Taxon DNA [53]. We evaluated the origin of monophyletic by tree-based analysis to access the effectiveness of marker discriminatory performance. Phylogenetic analyses were performed using maximum likelihood (ML) and Bayesian (BI). ML analyses were performed by RAxML-HPC BlackBox v.8.1.24 [60] at the CIPRES Science Gateway website [61] (http://www.phylo.org). For ML phylogenetic tree analyses, the best-fit models and general time reversible (GTR) + G were used with 1 000 bootstrap replicates. BI was performed with MrBayes 3.2 [62]. The BI model is constructed, and ngen is set to 1 000 000 burnin = ngen*0.25/ Samplefreq, Lset nst = 6 rates = invgamma, Prset statefreqpr = dirichlet (1, 1, 1, 1), graphical visualization of STRUCTURE results using Clumpak. Phylogenetic trees were visualized using Tree view. The best scoring tree was visualized with FigTree 1.3.1 (http://tree.bio.ed.ac.uk/).

## Supplementary Information

**Table S1.** Hypervariable region primer design.22 pairs of primers were designed using the software Geneious 1.10.

**Table S2.** Information about tested Lilium species in this study.

## Data Availability

The chloroplast genome sequences of 12 *Lillium* referred in this work were downloaded from the GenBank of NCBI (https://www.ncbi.nlm.nih.gov/). The sequences were proofreading using Geneious’s MAFFT v7 (http://mafft.cbrc.jp/alignment/server/). All the DNA barcoding-Seq raw data are available at NCBI, and accession numbers were listed in Table S2. The PHASE operation was performed with DNA SP v5.1 to calculate the index. The ML Tree was performed by RAxML-HPC BlackBox v.8.1.24 at the CIPRES Science Gateway website (http://www.phylo.org). The BI Tree was performed with MrBayes 3.2. The tree was visualized with FigTree 1.3.1 (http://tree.bio.ed.ac.uk/). Plant material was collected from the National Lily Germplasm Bank at Beijing Academy of Agricultural and Forestry Sciences.

## Abbreviations

*ITS*: Internal Transcribed Spacer
*rbc**L*: ribulose 1,5-bisphosphate carboxylase
*mat**K*: megakaryocyte-associated tyrosine kinase
*psbA-trnH*: Acer distylum PsbA- tRNA-His
*trnL-trnF*: tRNA-Leu- tRNA-Phe
*trnS-trnG*: tRNA-Ser- tRNA-Gly
*trnF-ndhJ*: tRNA-Phe-*ndhJ*
PCR: Polymerase Chain Reaction
cp genome: chloroplast genome
RAPD: Random Amplified Polymorphic DNA
ISSR: Inter-simple sequence repeat
Pi: the nucleotide polymorphism information
Eta: the total number of mutations
Hd: haplotype diversity
K: average nucleotide difference
K2P: Kimura-2-Parameter
ML: maximum likelihood
BI: Bayesian
