# Supplementary material for: Developing an efficient DNA barcoding system to differentiate between Lilium species
Source: BMC Plant Biol. 2021 Oct 13;21:465. doi: 10.1186/s12870-021-03229-6 (PMC8513328; doi:10.1186/s12870-021-03229-6)
Supplement: Supplementary file 1 — Table S1. Hypervariable region primer design.22 pairs of primers were designed using the software Geneious 1.10. [file 12870_2021_3229_MOESM1_ESM.doc]

*Table S1 Highly-variable region primer design*

| Forward primer | Reverse primer | Gene fragment | PCR Length | Region length |
| --- | --- | --- | --- | --- |
| LHV1-1/2F | LHV1-1R | *trnS-trnG* | 956 | 855 |
| LHV1-1/2F | LHV1-2R |  | 1078 | 855 |
| LHV2-1/2F | LHV2-1R | *trnE-trnT-psbD* | 839 | 881 |
| LHV2-1/2F | LHV2-2R |  | 843 | 881 |
| LHV3-1/2F | LHV3-1R | *trnF-ndhJ* | 909 | 628 |
| LHV3-1/2F | LHV3-2R |  | 997 | 628 |
| LHV4-1/2F | LHV4-1R | *psbE-petL* | 983 | 755 |
| LHV4-1/2F | LHV4-2R |  | 1109 | 755 |
| LHV5-1/3F | LHV5-1R | *trnP-psaJ-rpl33* | 716 | 665 |
| LHV5-2F | LHV5-2/3R |  | 734 | 665 |
| LHV5-1/3F | LHV5-2/3R |  | 1065 | 665 |
| LHV6-1/2F | LHV6-1R | *psbB-psbH* | 1346 | 1049 |
| LHV6-1/2F | LHV6-2R |  | 1123 | 1049 |
| LHV7-1/2F | LHV7-1R | *petD-rpoA* | 727 | 633 |
| LHV7-1/2F | LHV7-2R |  | 908 | 633 |
| LHV8-1/2F | LHV8-1R | *ndhF-rpl32-trnL* | 1575 | 1525 |
| LHV8-1/2F | LHV8-2R |  | 1720 | 1525 |
| LHV9-1F | LHV9-1/2 R | *ycf1a* | 1033 | 1124 |
| LHV9-2F | LHV9-1/2 R |  | 961 | 1124 |
| LHV9-3F | LHV9-1/2 R |  | 1310 | 1124 |
| LHV10-1F | LHV10-1/2R | *ycf1b* | 1078 | 710 |
| LHV10-2F | LHV10-1/2R |  | 914 | 710 |
